# Biochemical evolution in response to intensive harvesting in algae: Evolution of quality and quantity

**DOI:** 10.1111/eva.12632

**Published:** 2018-05-01

**Authors:** Dustin J Marshall, Rebecca J Lawton, Keyne Monro, Nicholas A Paul

**Affiliations:** ^1^ Centre for Geometric Biology/School of Biological Sciences Monash University Melbourne Vic. Australia; ^2^ MACRO—the Centre for Macroalgal Resources and Biotechnology James Cook University Townsville QLD Australia; ^3^ Bay of Plenty Regional Council Mount Maunganui New Zealand; ^4^ Faculty of Science, Health, Education and Engineering University of the Sunshine Coast Maroochydore QLD Australia

**Keywords:** crop production, experimental evolution, harvesting

## Abstract

Evolutionary responses to indirect selection pressures imposed by intensive harvesting are increasingly common. While artificial selection has shown that biochemical components can show rapid and dramatic evolution, it remains unclear as to whether intensive harvesting can inadvertently induce changes in the biochemistry of harvested populations. For applications such as algal culture, many of the desirable bioproducts could evolve in response to harvesting, reducing cost‐effectiveness, but experimental tests are lacking. We used an experimental evolution approach where we imposed heavy and light harvesting regimes on multiple lines of an alga of commercial interest for twelve cycles of harvesting and then placed all lines in a common garden regime for four cycles. We have previously shown that lines in a heavy harvesting regime evolve a “live fast” phenotype with higher growth rates relative to light harvesting regimes. Here, we show that algal biochemistry also shows evolutionary responses, although they were temporarily masked by differences in density under the different harvesting regimes. Heavy harvesting regimes, relative to light harvesting regimes, had reduced productivity of desirable bioproducts, particularly fatty acids. We suggest that commercial operators wishing to maximize productivity of desirable bioproducts should maintain mother cultures, kept at higher densities (which tend to select for desirable phenotypes), and periodically restart their intensively harvested cultures to minimize the negative consequences of biochemical evolution. Our study shows that the burgeoning algal culture industry should pay careful attention to the role of evolution in intensively harvested crops as these effects are nontrivial if subtle.

## 1 INTRODUCTION

Humans have increasingly turned to high‐intensity cultivation approaches to meet ever increasing demands for food, natural products and biofuels. High‐yield harvesting regimes impose significant selection on morphology, life history and behaviour, and harvested populations sometimes evolve in response (Enberg, Jørgensen, Dunlop, Heino, & Dieckmann, 2009; Hendry et al., 2011; Law, 2000; Proaktor, Coulson, & Milner‐Gulland, 2007; Reznick & Ghalambor, 2005). In turn, these evolutionary responses can alter the productivity and sustainability of the harvested populations, sometimes in negative ways (Law & Salick, 2005; Walsh, Munch, Chiba, & Conover, 2006).

Biochemical composition may also respond to selection—a range of artificial selection studies show evolution in biochemistry, from plants through to insects and fish. One of the best known examples of responses to direct selection on biochemistry comes from the Illinois long‐term selection experiment, where more than 100 years of selection has altered the oil content of maize. Examples of biochemical evolution in response to indirect selection imposed by harvesting are rarer, however, and the results of the few such studies are mixed. For example, Redpath, Cooke, Arlinghaus, Wahl, and Philipp (2009) found no difference in lipid, protein or carbohydrate content between largemouth bass selected for different levels of vulnerability, despite marked changes in growth rates. In contrast, Moreno et al. (2016) found that selected lines of salmon showed major changes in amino acid composition relative to unselected wild types. Thus, the extent to which culture and harvesting induces biochemical changes in target populations remains unclear. This knowledge gap is particularly important given many harvested populations are targeted specifically for their biochemical properties, and if these properties change, then the value of the crop may also change.

Evolutionary changes in biochemistry in response to harvesting regimes are of particular interest in algal production, yet the potential for such changes has gone largely unexplored. The production of algae in intensive land‐based culture systems is a relatively new approach with significant commercial potential and interest, particularly with regard to algal biochemistry. Algae have been proposed as substrates for a broad range of biofuels (Brennan & Owende, 2010; Elliott, Biller, Ross, Schmidt, & Jones, 2015; Mata, Martins, & Caetano, 2010; Rowbotham, Dyer, Greenwell, & Theodorou, 2012), and as a source of new bioproducts and animal feed (Gosch, Magnusson, Paul, & Nys, 2012; Jiménez‐Escrig, Gómez‐Ordóñez, & Rupérez, 2012; Li, Wijesekara, Li, & Kim, 2011; Pulz & Gross, 2004). A key concern here is the productivity of biochemical components (lipid, protein, carbohydrate) per unit area of cultivation—whether this productivity evolves in response to intensive harvesting remains unclear.

What is typically proposed for the intensive production of algae in land‐based systems is the production of a single species through asexual propagation, or fragmentation followed by cell division. Such systems are characterized by the continuous culture of algae at high densities and periodic, incomplete harvesting of the standing stock (Capo, Jaramillo, Boyd, Lapointe, & Serafy, 1999; Mata, Magnusson, Paul, & de Nys, 2015; Moheimani & Borowitzka, 2006; Rodolfi et al., 2009). A key requirement for commercial algal production is that the per unit area production of desirable biochemical qualities of the focal species is maintained despite intensive harvesting. Yet, few studies have determined whether harvesting regimes induce evolution in algal biochemistry. Given harvesting is likely to impose strong selective pressures, evolutionary responses in algal biochemistry are likely and could quickly reduce productivity.

There is evidence that key elements of algal biochemistry are evolutionarily labile. For example, targeted artificial selection on biochemical components can induce change in lipids and carbohydrates (Hathwaik et al., 2015). Given the observed negative changes in life history and morphology that often evolve in response to intense harvesting more generally, it is possible that similar negative changes could occur during algal production. However, we are not aware of any study that has explored evolutionary responses in algal biochemistry to the indirect selection associated with high‐intensity harvesting regimes. As such, the risks such evolution poses to commercial operations remain unknown.

Here, we evaluate the evolutionary consequences of high‐yield harvesting regimes for the production of a freshwater green alga from the genus *Oedogonium* in intensive culture systems. *Oedogonium* is a cosmopolitan genus of filamentous freshwater green macroalgae that has a worldwide distribution and is a common component of natural ecosystems. It is an unbranched, uniseriate green alga made up of small cylindrical cells. *Oedogonium* is a robust and competitively dominant genus that has been identified as a target for the treatment of freshwater waste streams (Cole, de Nys, & Paul, 2014; Roberts, de Nys, & Paul, 2013) and as a feedstock biomass for bioenergy applications (Lawton, de Nys, & Paul, 2013; Neveux, Magnusson, Maschmeyer, de Nys, & Paul, 2014; Neveux, Yuen, et al., 2014). We created replicate lines for three *Oedogonium* strains to 12 weeks of different harvesting regimes. At the end of 12 weeks, all cultures from both treatments were maintained for a further 4 weeks under a medium‐yield common garden regime (Garland & Rose, 2009). In a previous analysis of the same experimental evolution experiment, we examined growth rates and biomass productivity (Lawton, Paul, Marshall, & Monro, 2017). We demonstrated that harvesting regimes generate evolutionary responses for some commonly measured growth metrics, but once this is standardized for the ash‐free dry biomass productivity (e.g., total productivity), total productivity is fairly similar between harvesting regimes. In that study however, we did not examine changes in algal biochemistry. From a practical perspective, the productivity (mass of bioproduct per unit area per unit time) of the desirable bioproducts is the most important metric (as opposed to concentration of bioproducts per unit biomass). Thus, in this study, we focus on the productivity rather than the concentration of the desirable bioproducts. The key biochemical components for algal production that are of commercial interest are oils (particularly omega‐3 fatty acids), as well as key amino acids such as lysine and methionine. In addition to these specific components, which can be extracted first and are the most lucrative constituents, the residual meal (mostly carbohydrate) can be used for bioenergy applications, such that the higher heating value of the whole biomass is an appropriate measure of energy potential. First, we examined the relative levels of energy and the proximate analyses of lipid, carbohydrate and protein and, then, separately compare the content of each component and the productivity (amount produced per unit area). Next, we examined the key biochemical components of particular interest to industry, including omega‐3 and omega‐6 polyunsaturated fatty acids and the key essential amino acids.

## 2 MATERIALS AND METHODS

### 2.1 Sample collection and isolation

This experiment used three strains of *Oedogonium*—Tsv1, Tsv2 and Tsv11—that were originally isolated from samples collected from naturally occurring water bodies and wetland areas around Townsville, Queensland, Australia (Lawton, de Nys, Skinner, & Paul, 2014; Appendix S1). All three strains are genetically distinct from each other (Lawton et al., 2014). Tsv2 was identified as *O. intermedium* using taxonomic keys (Entwisle, Skinner, Lewis, & Foard, 2007). It was not possible to identify Tsv1 or Tsv11 to species level as specific morphological characteristics were not visible in either strain and their ITS sequences did not match the ITS sequences of any *Oedogonium* species in Genbank (Lawton et al., 2014, Appendix S1). Following isolation, strains were maintained in nutrient‐enriched autoclaved freshwater in a temperature and light controlled laboratory (12:12 light:dark cycle, 50 μmol photons m^−2^ s^−1^, 23°C) at James Cook University (JCU) for at least 2 years prior to the experiment. Stock cultures of each strain were established in 1000‐L plastic tanks maintained in a glasshouse with ambient natural light at the Marine and Aquaculture Research Facility Unit, James Cook University. Each culture strain was founded from a single filament so all genetic variation came from de novo mutations from the original wild type. Cultures were provided with aeration by a continuous stream of air entering the cultures through multiple inlets around the base of the tanks. Stock cultures were maintained for 3 weeks under these conditions prior to the start of the experiment.

Nutrient enrichment in the laboratory maintenance conditions was the same as that used under experimental conditions (0.05 g/L enrichment with MAF growth medium, Manutech Pty Ltd, 13.4% N, 1.4% P). Densities in the laboratory maintenance conditions varied somewhat over time, but were maintained around a long‐term average of 1 g FW/L. This is the same density that outdoor stock cultures of each strain were maintained during the 3‐week period prior to the start of the experiment and that cultures were maintained under during the four cycles of common garden in the last 4 weeks of the experiment.

### 2.2 Selection experiment

Twenty replicate monocultures of each strain containing equal quantities of fresh weight (FW) biomass were established. Ten replicate cultures of each strain were randomly assigned as high‐yield harvest, and 10 replicate cultures were randomly assigned as low‐yield harvest. High‐yield and low‐yield harvest treatments were created by resetting the stocking density of cultures at each harvest to a specified value. The high‐yield harvest cultures were stocked at an initial density of 0.5 g FW/L, and low‐yield harvest cultures were stocked at an initial density of 2 g FW/L. These stocking densities were chosen based on average growth rates recorded in our production system and data from a growth curve experiment (Lawton et al., 2017). These stocking densities mean that approximately 70% of the biomass is removed at each harvest for the high‐yield harvest treatment and 20% of the biomass is removed for the low‐yield harvest treatment.

Cultures were grown in 20‐L plastic buckets in a glasshouse with ambient natural light at the Marine and Aquaculture Research Facility Unit, JCU. Culture water was enriched (0.05 g/L) with MAF growth medium (Manutech Pty Ltd, 13.4% N, 1.4% P). Buckets were placed in a water bath with continuous flow to minimize large temperature fluctuations. Average water temperature throughout the experiment was 25.6°C (±0.7 *SD*), and cultures received an average total photosynthetically active radiation of 150 mol photons m^−2^ week^−1^ (±33 *SD*). Cultures were provided with aeration by a continuous stream of air entering the cultures through multiple inlets around the base of the buckets.

Every 7 days, each culture was harvested, and the biomass was briefly spun in a centrifuge to remove excess water and then weighed to determine the FW. The same biomass was then restocked back into each culture, with stocking density reset back to the relevant treatment level (0.5 or 2 g FW/L) by removing excess biomass. Biomass from each culture was kept separate and never mixed, enabling maintenance of a pure “line” of biomass throughout the experiment. Due to the fast vegetative (clonal) growth of *Oedogonium* filaments in intensive culture systems, the biomass restocked into cultures following harvesting each week can be considered to be a new clonal “generation” (Lawton, Carl, de Nys, & Paul, 2015). All excess biomass from each culture not used for restocking was weighed to determine the FW, dried in an oven at 65°C for at least 48 hr and then reweighed to determine the fresh weight:dry weight ratio (FW:DW) for each individual culture. This entire process was repeated every week for a total of 12 weeks, resulting in 12 cycles of differential selection (high‐yield/low‐yield harvest treatments). The process was then repeated for a further 4 weeks, with stocking densities of all cultures reset to 1 g FW/L following harvesting. These 4 weeks represent four cycles of common garden conditions. A stocking density of 1 g FW L is equivalent to a medium‐yield harvesting treatment and means that approximately 50% of the biomass is removed at each harvest. Thus, the maximum possible proportion of algal cells that could be retained from the 12‐week differential selection period after common garden 4 weeks was 6.25% and this assumes that individual algal cells live for longer than 4 weeks. Overall then, any persistent differences in mean phenotype between selection regimes after 16 weeks can be attributed to genetic change rather than residual environmental effects.

The experiment was run from March to April 2015 in North Queensland, Australia (19.26°S, 146.82°E). This time period corresponds to the end of the tropical wet season and the start of the dry season. During this time of year, the day length is around 12 hr.

### 2.3 Cell morphology

Cell volume was analysed in three randomly chosen replicate cultures from each strain x treatment combination, based on small samples of excess biomass that had been preserved in Lugol's solution (1%) at week 12 (end of differential selection) and week 16 (end of common garden selection). Four replicate subsamples of each preserved sample were viewed under a compound microscope (Olympus model BX53) at 20× magnification. The width and length of a single cell were measured using Olympus cellSens software (V. 1.7) on each of five replicate branches per subsample, and cell volume calculated from these measurements as π*r* ^*2*^ (where *r* was half the cell width).

### 2.4 Biochemical analyses—comparison of differential harvesting and common garden

Dried biomass (three randomly chosen replicate cultures from each treatment and strain combination) at the end of differential selection (week 12), and the end of common garden selection (week 16 of the selection experiment) was analysed for carbon, hydrogen, oxygen, nitrogen, sulphur and phosphorus (ultimate analysis), ash content and total lipid, protein and carbohydrate content.

To examine temporal changes in biochemical properties from the beginning of the experiment to the end, we had planned to use dried week 0 samples as a baseline but a storage error meant that these samples were unavailable. Thus, we used dried biomass samples from week 1 in their stead on the assumption that few biochemical changes had occurred within 1 week, particularly as a large proportion of biomass in each sample would have been present at week 1. Nevertheless, we acknowledge that the week 1 samples are an imperfect estimate of the original condition as some change must have occurred. From a practical perspective, temporal changes in biochemistry are inevitable due to differences in culture conditions, and seasonal changes in temperature and light (our experiments were carried out outdoors so as to be as realistic as possible). Producers are not interested in temporal changes in phenotypes per se, rather how different harvesting regimes alter the productivity of desirable bioproducts and so our main comparisons of interest are the differences among harvesting regimes at the end of the experiment.

There was not sufficient biomass to allow analysis of all biochemical characteristics for some replicate cultures. In these cases, biomass was analysed for ash, CHONPS and total lipids in order of priority until no biomass was left and another replicate culture from the same treatment was used for the remaining analyses. Ultimate analysis was outsourced to OEA laboratories ( http://www.oealabs.com/), while % oxygen was calculated as %O = 100−∑(C, H, N, S, ash) where C, H, N, S and ash are expressed as a percentage of the total mass. Ash content was determined by combusting a 100–300 mg subsample of dried biomass at 550°C in a muffle furnace until constant weight was reached. Total lipid content (% DW) was determined as described in Gosch et al. (2012), while protein was calculated based on the ultimate analysis of nitrogen content (% DW) of the biomass multiplied with a protein to nitrogen factor of x 4.7 (Neveux, Magnusson, et al., 2014), and carbohydrate was calculated by difference as 100−∑(lipid, protein, ash) where lipids, proteins and ash are expressed as a percentage of the total weight. The carbohydrate, protein and lipid productivities (g DW m^−2^ day^−1^) of each strain were calculated for each treatment by multiplying the DW productivity of each replicate from week 12 and 16 of the experiment by its carbohydrate, protein or lipid content (% DW).

To quantify the suitability of the biomass as a potential energy feedstock, the higher heating value (HHV) was calculated for each sample using the concentrations of carbon, hydrogen, oxygen, nitrogen and sulphur calculated as described above. The HHV is based on the elemental composition of the biomass and is a measure of the amount of energy stored within. The HHV was calculated using the equation HHV (MJ/kg) = 0.3491*C + 1.1783*H + 0.1005*S−0.1034*O−0.0151*N−0.0211*ash, where C, H, S, O, N and ash are the carbon, hydrogen, sulphur, oxygen, nitrogen and ash mass percentages of the algae on a dry basis (Channiwala & Parikh, 2002). The energy productivity (MJ m^−2^ day^−1^) was calculated for each treatment by multiplying the DW productivity (converted to kg DW m^−2^ day^−1^) of each replicate from week 12 and 16 of the experiment by its HHV (MJ/kg).

### 2.5 Biochemical analyses—fatty acid and amino acid comparisons after common garden selection

The fatty acid composition and amino acid composition of biomass at the end of common garden regime (week 16) were quantified for the same three replicate cultures chosen for biochemical analyses above. Fatty acid composition and amino acid composition were only quantified in samples taken at the end of common garden phase to determine whether there were long‐term effects of selection on individual metabolites that may have been masked by the proximate analysis. A direct transesterification method was used to simultaneously extract and esterify the fatty acids to fatty acid methyl esters (FAMEs) for analysis by gas chromatography mass spectrometry (GC–MS; 7890A GC, 5975C MS, DB‐23 capillary column with 15 μm cyanopropyl stationary phase, 60 m length and 0.25 mm inner diameter (Agilent Technologies Australia Pty Ltd.), as described in detail in Gosch et al. (2012). The content of total fatty acids (TFA) was determined as the sum of all FAMEs with fatty acids being designated as CX:Y(n‐z), where X is the total number of carbon, Y is the number of double bonds, and z is the position of the ultimate double bond from the terminal methyl group. In our previous work, we showed that there are sometimes quantifiable effects on the content or relative growth rates due to harvest, but these effects are not detected at the system level of cultures (Lawton et al., 2017). Therefore, we also formally analyse the fatty acid productivities (g DW m^−2^ day^−1^; a system production metric) of each strain, which were calculated for each treatment by multiplying the DW productivity of each replicate from week 12 to 16 of the experiment by its fatty acid content (mg/g algal DW).

Amino acids were analysed after liquid hydrolysis in 6 M HCl for 24 hr at 110°C using a Waters ACQUITY UPLC at the Australian Proteome Analysis Facility, Macquarie University, Sydney, using procedures based on the Waters AccQTag amino acid methodology (Bosch, Alegría, & Farré, 2006; Cohen & Fong, 2004). All cultures were analysed for aspartic acid, asparagine, glutamic acid, glutamine, serine, histidine, glycine, threonine, alanine, arginine, tyrosine, valine, methionine, phenylalanine, isoleucine, leucine, lysine and proline. As asparagine is hydrolysed to aspartic acid and glutamine to glutamic acid during analysis, the sum of these amino acids was reported as asparagine/aspartic acid or glutamic acid/glutamine. Cysteine tryptophan and taurine were not analysed as these require different analytical methods and represent only a very small fraction (<2%) of the total amino acids present in green macroalgae (Angell, Mata, de Nys, & Paul, 2014; Angell, Pirozzi, De Nys, & Paul, 2012). The amino acid productivities (g DW m^−2^ day^−1^) of each strain were calculated for each treatment by multiplying the DW productivity of each replicate from week 12 and 16 of the experiment by its amino acid content (mg g algal/DW).

For all our analyses, clonal line was the unit of replication where strain and treatment were fixed effects. We analysed the various biochemical constituents at week 12 and week 16 separately because we used much more detailed biochemical analyses in week 16 relative to week 12.

## 3 RESULTS

### 3.1 Proximate components and energy

**Figure 1:**
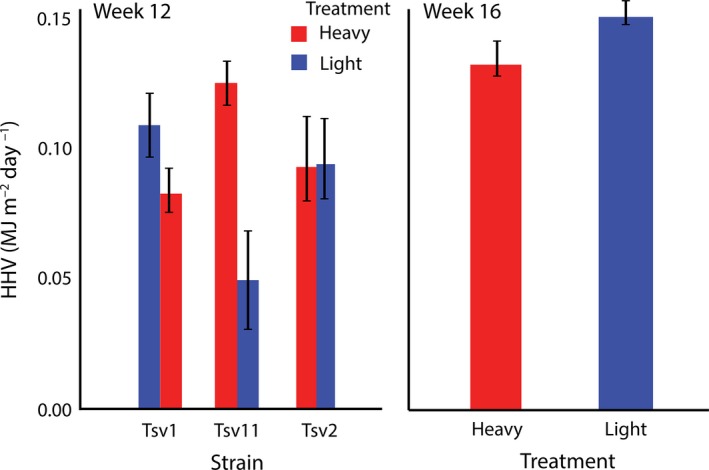
Higher heating value (HHV) (means ± *SE*) of three different strains after 12 weeks of differential selection (left panel) and after 12 weeks of differential selection plus 4 weeks of common garden selection (right panel) in *Oedogonium*

**Figure 2:**
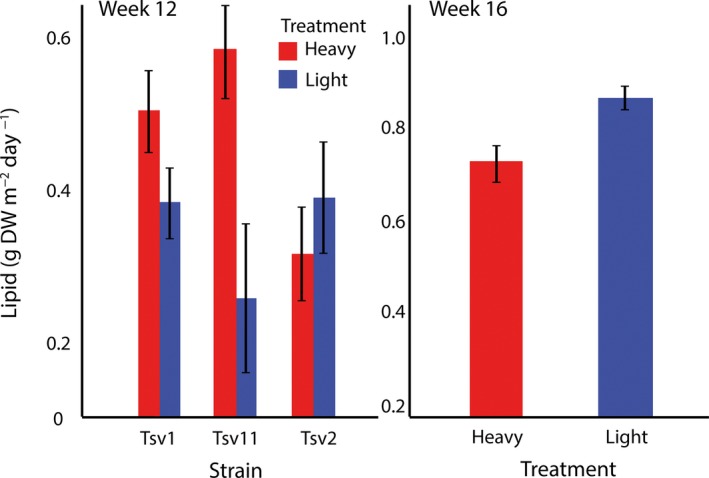
Lipid values of three different strains (means ± *SE*) after 12 weeks of differential selection (left panel) and after 12 weeks of differential selection plus 4 weeks of common garden selection (right panel) in *Oedogonium*

**Figure 3:**
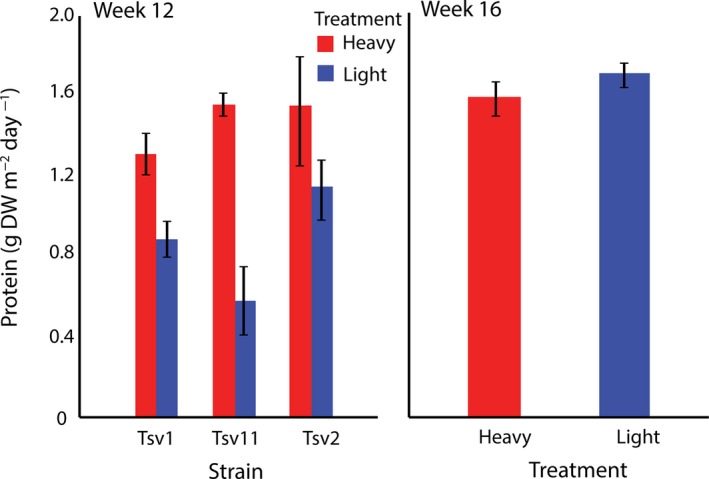
Protein values (means ± *SE*) of three different strains after 12 weeks of differential selection (left panel) and after 12 weeks of differential selection plus 4 weeks of common garden selection (right panel) in *Oedogonium*

After 12 weeks of the harvesting selection regime, there were strong interactions between strain and harvesting regime on higher heating value (HHV) and lipids. HHV and lipids were higher in the heavy harvesting regime for strains Tsv1 and Tsv11 relative to the light harvesting regime, but there was no difference for Tsv2 (Table 1; Figures 1 and 2). There was a strong effect of harvesting regime on protein across all three strains, however, with higher productivity of protein in the heavy harvest regime (Figure 3). Relative to levels at week 1 of harvesting, carbohydrates tended to similar or lower than original levels (depending on the harvesting regime), HHV was unchanged and lipids and proteins tended to be lower (Table 2). These temporal changes could be due to the experimental conditions or seasonal variation.

**Table 1:** Effect of harvesting regime on the productivity of higher heating value (HHV), lipids and protein in *Oedogonium* after differential selection (12 weeks)

Component	Source	*df*	*F*	*p*
HHV	Strain × Treat	2	4.95	**.022**
Lipid	Strain × Treat	2	6.52	**.009**
Protein	Strain	2	1.09	.360
	Treat	1	12.93	**.002**
	Strain × Treat	2	1.46	.264
	Error	15		

Significant differences indicated in bold.

**Table 2:** Comparisons of productivities (grams dry weight m^−2^ day^−1^) of *Oedogonium* cultures for various coarse bioproducts across different weeks and selection regimes. Final two columns show ratios of productivity of week 1 vs week 12 and 16

Component	Selection	Week			Ratio	
		1	12	16	12:1	16:1
Protein	Heavy	22.5	23	24	1.03	1.07
	Light	21.1	27	26	1.28	1.23
Lipid	Heavy	10.2	8.4	11.1	0.82	1.09
	Light	10.3	9.5	12.2	0.91	1.18
HHV	Heavy	19.2	19.6	19.8	1.02	1.03
	Light	19.2	19.7	20.2	1.02	1.05
Carbohydrate	Heavy	62.9	55.2	54.3	0.87	0.86
	Light	60.4	61.0	58.1	1.00	0.96

Once the selection lines were placed in the common garden harvesting regime for 4 weeks (week 16), the effects of the 12‐week harvesting period largely reversed (Figure 1, 2 and 3). The harvesting regime x strain interactions dissipated for all components, leaving only a main effect of harvesting regime for lipid (Table 3). In contrast to week 12, where heavy harvesting tended to have higher values (in two of three strains), once in a common garden regime, heavy harvesting selected lines had lower lipid productivity than light harvesting selected lines (Figure 2). Lipid levels in both heavy and light regimes were slightly higher than earlier (week 1) levels, but again it is unclear whether these changes were associated with seasonal effects or experimental condition. Similar, though nonsignificant trends were observed for HHV and carbohydrate with slight decreases in productivity in the heavy harvest regime relative to the light regime (Figure 1 and 3). Relative to earlier levels, carbohydrate decreased markedly, while HHV remained at similar levels (Table 2). Protein, while not significantly different between harvesting regimes, was higher in the light harvesting regime relative to earlier levels but largely unchanged in the heavy regime.

**Table 3:** Effect of harvesting regime on the productivity of higher heating value (HHV), lipids and protein in *Oedogonium* after common garden regime (4 weeks of identical selection following 12 weeks of differential selection)

Component	Source	*df*	*F*	*p*
HHV	Strain	2	0.80	.469
	Treat	1	3.13	.099
	Strain × Treat	2	0.30	.745
	Error	14		
Lipid	Strain	2	5.97	**.013**
	Treat	1	12.48	**.003**
	Strain × Treat	2	0.80	.470
	Error	14		
Protein	Strain	2	3.57	.056
	Treat	1	0.10	.757
	Strain × Treat	2	0.36	.708
	Error	14		

Significant differences indicated in bold.

### 3.2 Fatty acids and amino acids

**Figure 4:**
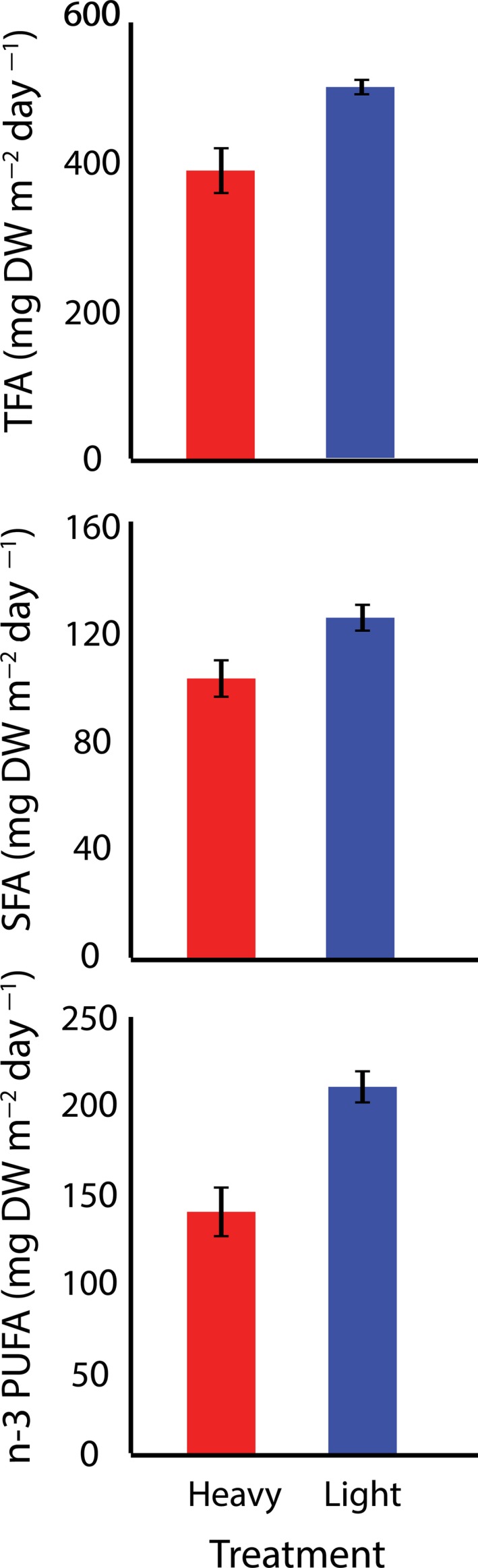
Fatty acid (FA) (means ± *SE*) content of *Oedogonium* under differential selection regimes after 12 weeks of differential plus 4 weeks of common garden selection. *n*‐3 PUFA, polyunsaturated omega‐3 fatty acids; SFA, saturated fatty acids; TFA, total fatty acids

**Figure 5:**
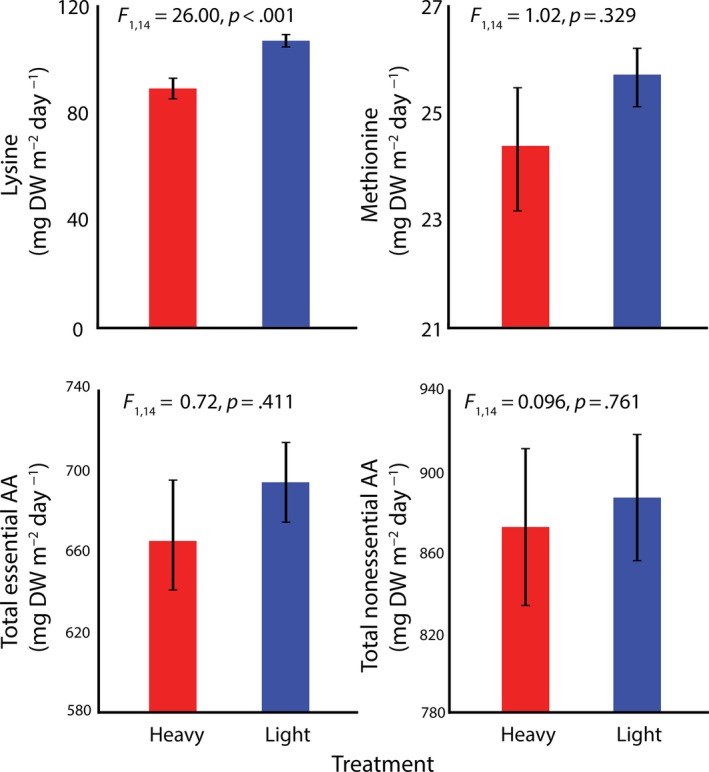
Amino acid (AA) content (means ± *SE*) of *Oedogonium* under differential selection regimes after 12 weeks of differential plus 4 weeks of common garden selection

Specific components in the selected lines showed persistent changes in response to the selection regime after selection had been relaxed (Table 4). Generally, the productivity of desirable bioproducts decreased in the heavy harvested regime, particularly for fatty acids but also for lysine (Figures 4 and 5).

**Table 4:** Analyses of amino acid (AA) and fatty acid (FA) productivity after common garden regime (4 weeks of identical selection following 12 weeks of differential selection) across strains of *Oedogonium*

Component	Source	*df*	*F*	*p*
Lipid				
Total FA	Strain	2	3.51	.058
	Treat	1	11.76	**.004**
	Strain × Treat	2	0.51	.614
	Error	12		
Saturated FA	Strain	2	7.89	**.005**
	Treat	1	7.29	**.017**
	Strain × Treat	2	0.01	.987
	Error	12		
MUFA	Strain	2	7.02	**.008**
	Treat	1	0.94	.350
	Strain × Treat	2	2.97	.089
	Error	12		
PUFA	Strain	2	3.61	.054
	Treat	1	19.12	**.001**
	Strain × Treat	2	1.35	.297
	Error	12		
Omega‐3	Strain	2	24.34	**<.001**
	Treat	1	63.55	**<.001**
	Strain × Treat	2	0.42	.667
	Error	12		
** **Omega‐6	Strain	2	4.22	**.037**
	Treat	1	1.18	.296
	Strain × Treat	2	2.60	.116
	Error	12		
Protein				
Nonessential AA	Strain	2	2.04	.167
	Treat	1	0.10	.761
	Strain × Treat	2	1.08	.371
	Error	12		
Essential AA	Strain	2	3.82	**.047**
	Treat	1	0.72	.411
	Strain × Treat	2	0.28	.763
	Error	12		
Methionine	Strain	2	3.89	.686
	Treat	1	1.02	.329
	Strain × Treat	2	0.40	.677
	Error	12		
Lysine	Strain	2	4.98	**.023**
	Treat	1	26.00	**<.001**
	Strain × Treat	2	3.25	.074
	Error	12		

MUFA, monounsaturated fatty acids; PUFA, polyunsaturated fatty acids.

Significant differences indicated in bold.

For omega‐3 fatty acids, as well as the aggregate total fatty acids, polyunsaturated and saturated fatty acids, the effect of harvesting regime was consistent with similar levels of reduced productivity of all in the heavy harvest regime relative to the light harvest regime (Figure 4).

For the amino acids, algae in the heavy harvest regime produced less lysine compared to algae in the light harvest regime, regardless of strain (Table 4, Figure 5). For the rest of the amino acids, there were indications of strain‐specific effects but these were not definitive nor were they consistent among different amino acids.

### 3.3 Cell volume

Cell volume differed slightly though not significantly between the harvest treatments at the end of differential selection (week 12; *F*_1,12_ = 3.66, *p* = .08). These differences were inconsistent among strains (Strain x Treat: *F*_2,12_ = 3.42, *p* = .066) and strains differed much more regardless of treatment (Strain: *F*_2,12_ = 173.5, *p* < .001). At this time, cell volume was larger in the low‐yield treatment compared to the high‐yield treatment for Tsv1, and Tsv11, but marginally smaller in the low‐yield treatment compared to the high‐yield treatment for Tsv2. By the end of the common garden treatment (week 16), only a difference between strains remained (Treat: *F*_2,12_ = 111.04; *p* < .001).

## 4 DISCUSSION

We found that harvesting intensity generated changes in the biochemistry of an alga of commercial importance (summarized in Table 5). While the two harvesting regimes generated biochemical differences between our lines, the nature of these changed once the lines were placed in the same, common garden regime. These changes suggested that the differences in environmental conditions during harvesting temporarily masked the underlying evolutionary responses to selection. Generally, algae grown under the heavy harvest regime had much lower lipid production once placed into a common garden regime. Our results suggest that different harvesting regimes alter the evolutionary trajectories of algae and heavy harvesting reduces the yield of desirable products. Although undesirable from an applied perspective, these evolutionary changes were largely in accordance with what might be expected from a general life‐history perspective and our understanding of algal biology. Importantly, modifications to existing large‐scale culture approaches should mitigate or reduce these undesirable impacts.

**Table 5:** Summary of effects of harvesting regime on strain‐specific and main effects on *Oedogonium* biochemistry and cell morphology after common garden regime (4 weeks of identical selection following 12 weeks of differential selection)

Component	Strain × Treatment	Treatment	Heavy harvesting	Light Harvesting
HHV	No	No		
Lipid	No	Yes	↓	↑
Total FA	No	Yes	↓	↑
Saturated FA	No	Yes	↓	↑
MUFA	No	No		
PUFA	No	Yes	↓	↑
Omega‐3	No	Yes	↓	↑
Omega‐6	No	No		
Protein	No	No		
Nonessential AA	No	No		
Essential AA	No	No		
Methionine	No	No		
Lysine	No	Yes	↓	↑
Cell volume	No	No		

MUFA, monounsaturated fatty acids; PUFA, polyunsaturated fatty acids.

Arrows indicates values significantly lower in the heavy harvesting regime. See Supplementary figures for graphical representation of fatty acid (FA) and amino acid (AA).

Both the heavy and light harvesting regimes changed relative to earlier (week 1 levels), but interpreting the drivers of these temporal changes is difficult. Because we conducted our study under realistic conditions in the open, the temperature and light regime varied over the course of the experiment, and both have known effects on algal phenotypes. Thus, any temporal changes across both treatments could simply reflect environmental variation. On the other hand, some effects could be driven by the experimental conditions common to both harvesting regimes relative to the original stock conditions from which algae we sourced. Thus, we cannot definitively say that heavy harvesting reduced productivity rates of desirable bioproducts relative to the original phenotype; rather, we must restrict all of our comparisons between the different harvesting regimes at any one point in time. Importantly, this is the comparison of most practical relevance to aquaculture as producers need to understand the consequences of different harvesting regimes.

The two harvest regimes created very different selection pressures on our study species. We already know that the heavy harvest regime favoured rapid growth rates over the harvest cycle (Lawton et al., 2017). Any phenotypes that failed to proliferate between harvesting bouts were likely to be eliminated from the cultures through simple sampling effects—phenotypes in low abundance were unlikely to be retained in the nonharvested fraction used as starter stock after each harvest. Accordingly, we have previously shown that specific growth rates evolved to be faster in the heavy harvest regimes (Lawton et al., 2017). It is possible that the heavy harvest regime favoured a “live fast” phenotype relative to the light harvest regime, but growth rate was not the only trait that was likely to be under selection.

The two harvest regimes generated very different resource conditions, both in terms of mean and variance. In the heavy harvest regime, a large proportion of biomass was removed each time, such that the mean ratio of resources (both nutrients and light) to biomass was much greater in the heavy harvest regime relative to the light harvest regime. This difference in ratios arises because the heavy harvest cultures were reduced to a much lower biomass each stocking time, while the amount of resources we supplied to each culture remained unchanged (such conditions reflect standard culture practices). Furthermore, because populations under the two regimes attained very similar biomasses just before harvesting, the range of biomass:resource ratios experienced by the heavy harvest lines was much greater than the range of ratios experienced by the lighter harvest lines. We will now consider our results in the light of these differences in resources.

We found that under heavy harvesting regimes, where resources (both light and nutrients) were both more abundant and more variable and faster growth was favoured, the protein content of algae was higher after 12 weeks of selection. This phenotypic difference in protein matches other studies of algae, where algae grown under higher nitrogen conditions tend to have higher protein contents (Renauld et al., 1991; Uttin, 1985). Once the differences in culture conditions were removed under the common garden regime, the algae under both regimes had similar levels with regard to protein. However, the placement into common garden conditions revealed evolutionary difference that persisted for one amino acid (lysine) and most fatty acids. Importantly, these shifts were undesirable from a commercial perspective.

Our results suggest that industrial applications of algal culture face a paradox. On the one hand, intensive harvesting leads to more rapid growth rates, higher protein productivity (at least initially) and higher biomass yields overall. On the other hand, productivity of other desirable products (fatty acids, lysine) goes down. The apparent benefit of the heavy harvesting regime for protein productivity is probably driven by the fact that these cultures periodically experience higher nutrient regimes (immediately following a heavy harvest). These effects mask a gradual decline in lipid productivity relative to the light harvesting regime that only manifests once the different cultures are grown under the same conditions—it is likely that these differences would have eventually appeared even under the differential harvesting regimes had we continued the experiment for even longer but we can only speculate in the absence of data. It seems that producers face a trade‐off between productivity of biomass and productivity of desirable products such as fatty acids and lysine. Importantly, if we had presented raw concentrations of desirable products (as opposed to productivity), we would have overestimated the impact of the intensive harvesting regime—concentrations of desirable products drop substantially relative to the light regime. However, because total productivity goes up with more intensive harvesting regimes, the productivity of some desirable products decreases by a less substantial margin. Nevertheless, given we observed significant drops in the productivity of desirable products in the short term, this suggests that heavy harvesting effects complicate commercial efforts.

Our experiment suggests that heavy harvesting selects for phenotypes that are less desirable from a commercial perspective, but our study also suggests a relatively practical solution to this issue. We found that cultures maintained at relatively high densities (the light harvest regime) retained their desirable qualities in terms of higher heating value, lipids, carbohydrates and protein (all were almost identical to earlier levels). As such, we suggest maintaining high‐density “mother cultures” to periodically restart harvest culture. Thus, producers should be able to maintain the more desirable phenotypes while maximizing production more generally. Fortunately, the use of mother cultures of many microalgae with high‐value products is the operational norm to minimize the effects of predation, fouling or disease.

The biochemical components of different *Oedogonium* strains often responded differently to harvesting. Once harvesting conditions were the same however, idiosyncrasies among strains subsided and resolved into consistent differences between the heavy and light harvesting regimes. This implies that selection induced both some transient and some relatively stable changes in biochemistry that could reflect a combination of nongenetic and genetic mechanisms, respectively. The goal of our experiment was to exploit the latter, based on considerable evidence of heritable variation (e.g., due to somatic mutation) arising within clonal lineages of diverse taxa during growth (Fagerström, Briscoe, & Sunnucks, 1998; Gill, Chao, Perkins, & Wolf, 1995; Whitham & Slobodchickoff, 1981). Intraclonal variation of this kind can accumulate remarkably rapidly in several groups of macroalgae (Meneses, Santelices, & Sanchez, 1999; Poore & Fagerström, 2000) and especially in fast‐growing, filamentous forms like *Oedogonium* (Lawton et al., 2015; Monro & Poore, 2009). Similar to our results, past efforts to select upon intraclonal variation in red macroalgae have yielded responses that were consistent among genotypes for some traits and genotype‐specific for others (Monro & Poore, 2009), suggesting that the mutational target size of traits determines the amount of variation that becomes available for selection or that genotypes/strains differ in the rate that variants accumulate. Nevertheless, past work has shown that clonal propagation can also generate persistent nongenetic effects on trait expression (Schwaegerle, 2005), analogous to parental effects in sexual organisms. Hence, it is possible that such effects contributed to the significant strain x treatment effects on biochemistry during the differential harvesting regimes, but were eventually lost from strains under common garden conditions.

We find evidence for evolutionary‐derived differences in key biochemical components in an algal species of commercial interest under different harvesting regimes. Whether such differences are likely to occur more generally remains unclear at this stage but given the rapidly growing interest in algal aquaculture and the intensive harvesting regimes to which they will be subjected, such evolutionary responses seem likely. As the optimization of large‐scale algal culture continues, we suggest that such programmes consider the role that evolutionary responses may play in altering yields.

## CONFLICT OF INTEREST

None declared.

## DATA ARCHIVING STATEMENT

The raw data underlying the main results are available from the Dryad Digital Repository: https://doi.org/10.5061/dryad.sg4hb67.
