# Supplementary figures and images for: Biochemical evolution in response to intensive harvesting in algae: Evolution of quality and quantity
Source: Evol Appl. 2018 May 1;11(8):1389–400. doi: 10.1111/eva.12632 (PMC6099826; doi:10.1111/eva.12632)

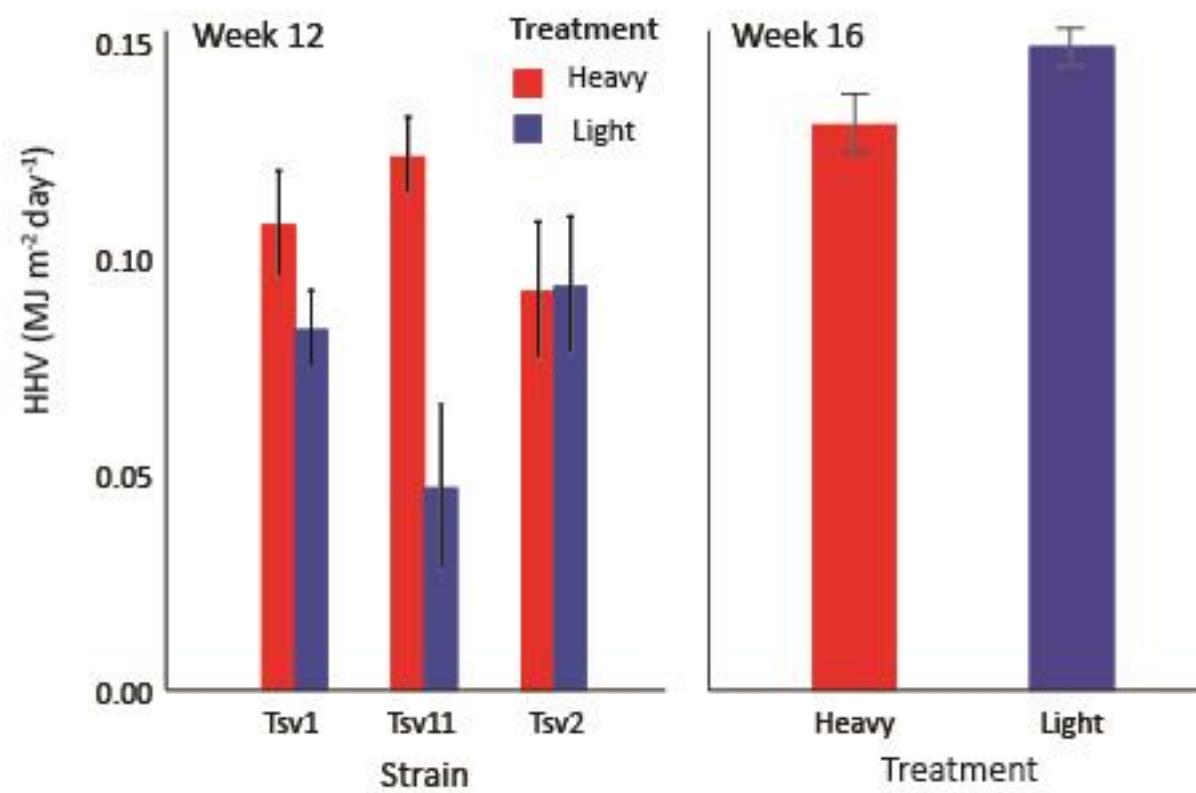

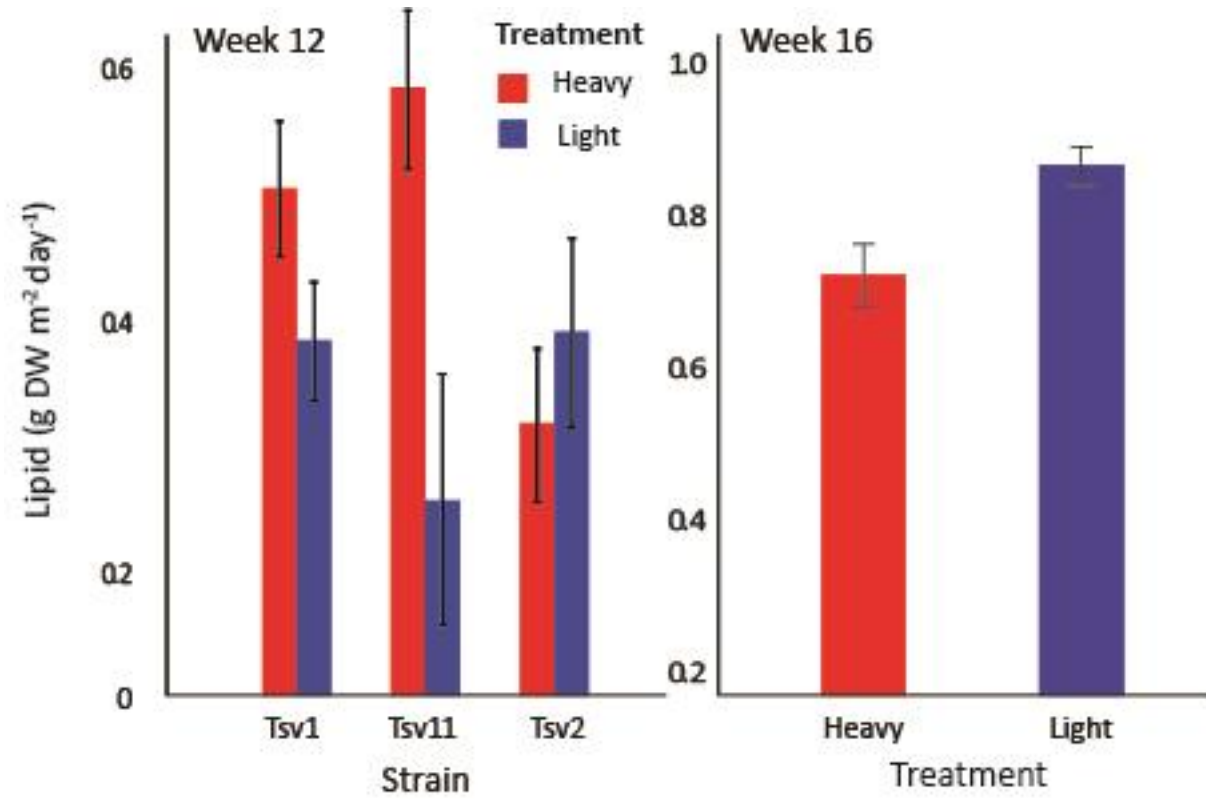

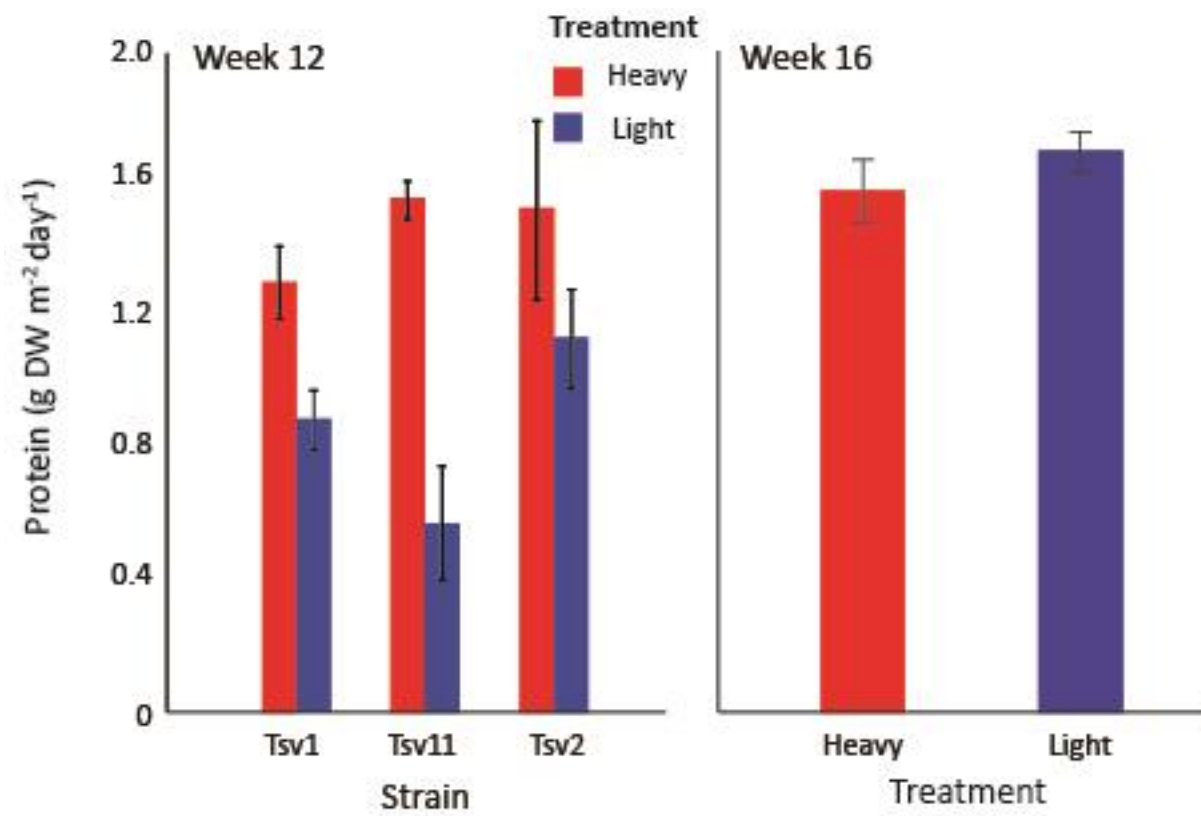

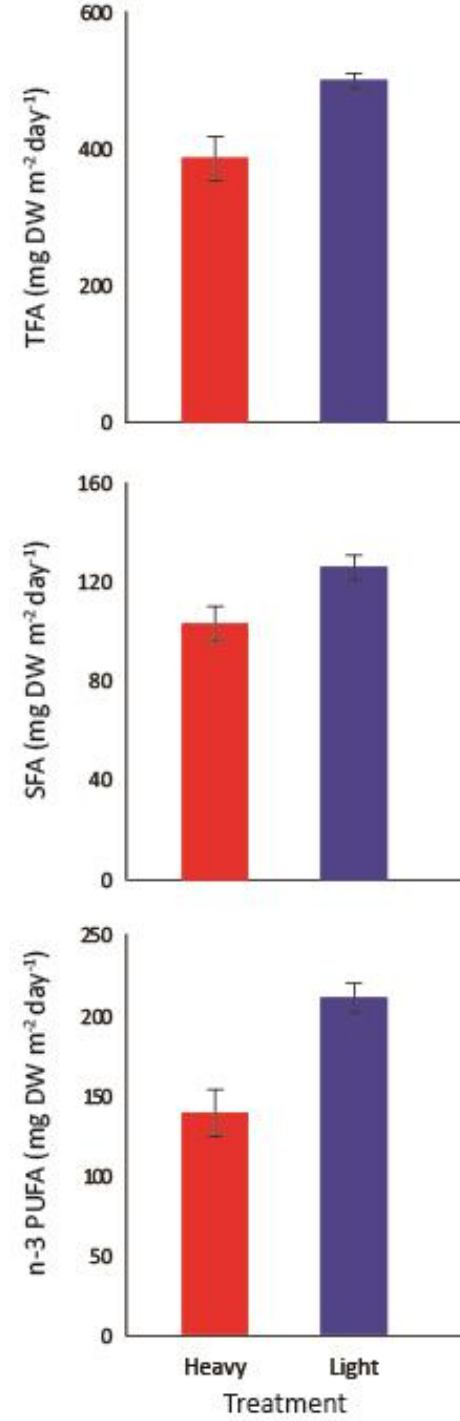

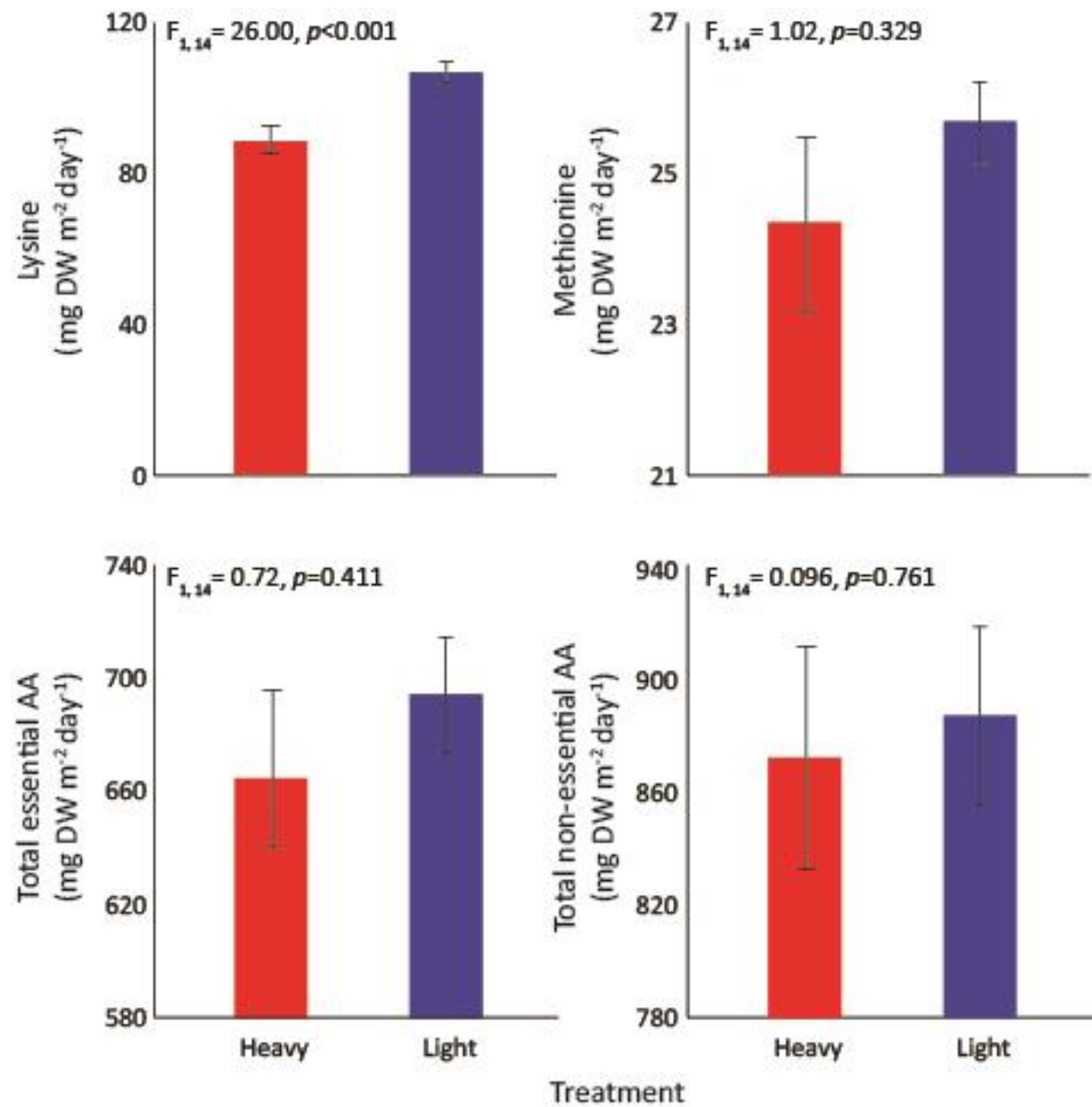

Supplement: Supplementary file 1 [file EVA-11-1389-s001.pdf]
